# Scaling Up Community-based Noncommunicable Disease Research into Practice in Pokhara Metropolitan City of Nepal (SCALE-NCD) trial: study protocol for a cluster-randomized controlled trial

**DOI:** 10.21203/rs.3.rs-8320113/v1

**Published:** 2026-05-25

**Authors:** Yoko Inagaki, Sweta Koirala, Maryam Hameed Khan, Kamal Ghimire, Pabitra Babu Soti, Niraj Bhattarai, Pratibha Bhandari, Chathurangi H Pathiravasan, Buna Bhandari, Rajshree Thapa, Khim Bahadur Khadka, Zhengbang Yao, Archana Shrestha, Vanessa Garcia-Larsen, Eric A. Finkelstein, Justin B. Moore, Svea Closser, Lawrence J. Appel, Per Kallestrup, Dinesh Neupane

**Affiliations:** Johns Hopkins Bloomberg School of Public Health; Nepal Development Society; Johns Hopkins Bloomberg School of Public Health; Johns Hopkins Bloomberg School of Public Health; Nepal Development Society; Nepal Development Society; Nepal Development Society; Johns Hopkins Bloomberg School of Public Health; Florida State University; Monash University; Health Directorate, Gandaki Province; Johns Hopkins Bloomberg School of Public Health; Kathmandu University; Johns Hopkins Bloomberg School of Public Health; Duke-NUS Medical School; Wake Forest University School of Medicine; Johns Hopkins Bloomberg School of Public Health; Johns Hopkins School of Medicine; Aarhus University; Johns Hopkins Bloomberg School of Public Health

**Keywords:** ypertension, diabetes, smoking, community health workers, mobile health

## Abstract

**Background:**

Rapid globalization and urbanization continue to escalate the burden of non-communicable diseases (NCDs) across the world, disproportionally affecting low- and middle-income countries (LMICs). To date, trials have documented that task-sharing with community health workers (CHWs) can reduce systolic blood pressure (SBP), reduce fasting blood glucose, and achieve smoking cessation. However, most studies have been conducted in rural settings, or focused on managing a single condition, such as hypertension only.

**Methods:**

We propose an open-label, two-armed, community-based cluster randomized controlled trial in Pokhara Metropolitan City of Nepal; the second largest city in Nepal. A total of 30 clusters will be randomized into intervention or control arm in a 1:1 ratio. An individual is eligible if living in Pokhara Metropolitan City and having one or more of the following conditions: hypertension, type 2 diabetes, and tobacco smoking. The participants will be recruited through study team home visits. The intervention group will receive an intervention package “SCALE-NCD” which includes 1) home-based monitoring, referral, and counseling for self-management of hypertension, diabetes and tobacco smoking by Female Community Health Volunteers (FCHVs), and 2) weekly mobile phone messages to promote healthy lifestyle. FCHVs are CHWs in Nepal whose usual tasks are limited to maternal and child health care services. The control group will receive usual care, where there is neither of aforementioned two components. Primary outcomes are change in SBP, change in fasting plasma glucose, and change in smoking cessation, the required sample size for which is 405, 105, and 525 per arm, respectively.

**Discussion:**

This study will inform us of the effectiveness of a FCHV-led, and home-based management of the top three NCD risk factors coupled with weekly mobile phone messages in urban Nepal. Building on the previous studies that measured the efficacy of FCHV-led home-based management of a single NCD risk factor in a small geographical area, this scaled-up study will provide us with the realistic impact that FCHVs may have if they are trained to provide primary care services for management of major NCD risk factors.

**Trial registration:**

ClinicalTrials.gov NCT06740708. Registered on 2024-12-13

## Administrative information

Note: the numbers in curly brackets in this protocol refer to SPIRIT checklist item numbers. The order of the items has been modified to group similar items (see http://www.equator-network.org/reporting-guidelines/spirit-2013-statement-defining-standard-protocol-items-for-clinical-trials/).

**Table:** 

Title {1}	Scaling Up Community-based Noncommunicable Disease Research into Practice in Pokhara Metropolitan City of Nepal (SCALE-NCD): study protocol for a cluster randomized controlled trial
Trial registration {2a and 2b}.	ClinicalTrials.gov NCT06740708 https://clinicaltrials.gov/study/NCT06740708
Protocol version {3}	This is version 13 based on the protocol submitted to Johns Hopkins Bloomberg School of Public Health Institutional Review Boards
Funding {4}	This project is supported by the National Heart, Lung, And Blood Institute of the National Institutes of Health under Award Number R01HL172271
Author details {5a}	Yoko Inagaki^1^, Sweta Koirala^2^, Maryam Hameed Khan^1^, Kamal Ghimire^1^, Chathurangi H. Pathiravasan^1^, Rajshree Thapa^3^, Buna Bhandari^4,5^, Khim Bahadur Khadka^6^, Niraj Bhattarai^2^, Pratibha Bhandari^2^, Zhengbang Yao^1^, Pabitra Babu Soti^2^, Archana Shrestha^7^, Vanessa Garcia-Larsen^1^, Eric A Finkelstein^8^, Justin P Moore^9^, Svea Closser^1^, Lawrence J Appel^1,10,11^, Per Kallestrup^12^, Dinesh Neupane^1, 11^ ^1)^Johns Hopkins Bloomberg School of Public Health, USA, ^2)^Nepal Development Society, Nepal, ^3)^Eastern Health Clinical School, Monash University, Australia ^4)^Florida State University College of Nursing, USA, ^5)^Department of Global Health and Population, Harvard T H Chan School of Public Health, USA ^6)^Health Directorate Gandaki Province, Nepal, ^7)^Kathmandu University School of Medical Sciences, Nepal, ^8)^Duke-NUS Medical School, Singapore, ^9)^Department of Implementation Science, Division of Public Health Sciences, Wake Forest University School of Medicine, USA ^10)^Johns Hopkins School of Medicine, USA ^11)^Welch Center for Prevention, Epidemiology and Clinical Research, USA, ^12)^Research Unit for Global Health, Department of Public Health, Aarhus University, Denmark
Name and contact information for the trial sponsor{5b}	Rebecca A Roper rebecca.roper@nih.gov
Role of sponsor {5c}	The funder had no role in the study design and will not be involved in data collection, management, analysis, interpretation, writing of the report, or the decision to submit the findings for publication. All aspects of the study will be carried out independently by the research team

## Introduction

### Background and Rationale {6a}

Non-Communicable Diseases (NCDs) are the leading cause of death and disability across the world. The World Health Organization (WHO) estimates that there are 44 million people die from NCDs each year, accounting for 75% of global deaths.^1^ The number of deaths from NCDs across the globe is projected to increase to 52 million by 2030.^2^ The burden of NCDs is disproportionately occur in low- and middle-income countries (LMICs); 86% of premature deaths from NCDs occur in LMICs, and people in LMICs have 1.5 times higher chance of dying from NCDs than those in high-income countries.^3,4^ As health systems in LMICs are less resourced and not adequately capacitated^5^, a clinically meaningful, cost-effective, and sustainable solution to improve prevention and control of NCDs at population level is much needed.

Nepal is a country with the lowest GDP per capita in South Asia^6^, and the burden of NCDs has rapidly increased over the past two decades as a result of globalization, urbanization, and population aging^7,8^. Global Burden of Disease 2021 reported that tobacco smoking (11.1%), high systolic blood pressure (SBP) (9.1%), and high fasting blood glucose (6.5%) are the top three risk factors contributing to all-cause deaths after air pollution (19.2%) in Nepal.^9^ The WHO Global Report on Hypertension documented that there are 4.8 million hypertensive adults living in Nepal, of which 36.0% were diagnosed, 20.0 % were under treatment with anti-hypertensive medicines, and only 9.0% had achieved the BP control goal of lower than 140/90 mmHg^10^. This is lower than the average for the LMICs (diagnosis 43.1%, pharmacological treatment 32.6%, and control 14.3%)^11^. Similarly, proportions of adults with type 2 diabetes diagnosed by physicians (26.5%), treated with oral hypo-glycaemic medicines (20.7 %), and achieving glycaemic control (6.0%) in Nepal^12^ is lower than the LMICs average.^11^

There are several factors that may contribute to poor control of common NCD risk factors such as hypertension and diabetes in Nepal. Conditions such as hypertension and diabetes do not typically exhibit symptoms in early stages. As a result, patients often seek care only after experiencing severe or fatal cardiovascular events, rather than attending regular check-ups. The other factors include lack of resources and skilled health providers who can screen, diagnose, initiate and titrate medical treatments. Nepal, like many other LMICs faces a severe shortage of physicians, a big proportion of whom migrate overseas for better paid opportunities^13^. Approximately 3 million hypertensive adults in Nepal are unable to receive even three annual physician visits due to the limited number of physicians relative to the growing patient demand.^14^ A wide in-country gap in physician density between urban (i.e. high density) vs rural (low density) areas poses another challenge to meeting the health service demand^15^. Additionally, essential commodities such as functioning diagnostic tools and medicines are not available in every primary healthcare facility, further limiting the ability of trained personnel to provide timely care to control NCDs.

One solution to improve the control of common NCD risk factors at the population-level is task-sharing with trained community health workers (CHWs). Task-sharing is an aspect of contemporary health systems in which some clinical tasks performed by physicians are implemented by trained non-physician health workers, thereby allowing physicians to allocate their time on the advanced tasks that require their specialized skills and knowledge.^16,17^ Task-sharing with trained CHWs has been of particular interest among researchers and policymakers because of their large workforce size^11^, ability to reach community population and identifying those who need medical services through door-to-door home visits, and their historical background in reducing maternal and child mortality through the Integrated Community Case Management (ICCM).^18^ To date, trials have indicated that sharing clinical tasks with trained CHWs can reduce systolic blood pressure^19,20^, fasting blood glucose^21–23^, and tobacco smoking^24,25^. In Nepal, in a cluster randomized control trial, it was demonstrated that sharing screening, referral, and self-management support (i.e. healthy lifestyle counseling) tasks with trained Female Community Health Volunteers (FCHVs); government certified CHWs, reduced mean systolic blood pressure (SBP) by 4.9 mm Hg (95%CI −7.78 to −2.00)^26^ and fasting blood glucose by 27.90 mg/dL (95% CI, −37.62 to −18.18)^27^ at 12-month follow-up. In another cluster randomized controlled trial, a greater smoking cessation rate was achieved among the intervention groups who received FCHVs-led lifestyle counseling to smoking cessation, compared to the control groups receiving usual care at 12 months.^28^

Another potential solution is the adoption of mobile health (mHealth) innovations. mHealth, defined as the use of mobile phones and digital technologies to enhance public health, offers significant opportunities to improve the prevention, diagnosis, and management of NCDs in low-resource settings. By facilitating real-time and remote communication, health education and monitoring, mHealth helps address barriers such as workforce shortages and limited access to healthcare facilities. Previous systematic reviews and meta-analyses have reported favourable outcomes from using text messaging (SMS) interventions for managing hypertension, including promoting lifestyle changes^29^ and improving medication adherence^30^. A cluster randomized controlled trial in Nepal also demonstrated that weekly SMS messages promoting self-management among hypertensive adults attending outpatient units of a tertiary hospital reduced SBP by 6.5 mmHg (95% CI: −12.6 to −0.33) over 12 months.

Despite the mounting evidence, there are still knowledge gaps to be filled. First, most of the existing trials tested the effectiveness of task-sharing with CHWs for the management of a single condition (e.g., hypertension only). As patients are likely to develop multiple NCD risk factors (e.g., hypertension and diabetes) through their life courses, there is a need to assess the effectiveness of task-sharing interventions in which CHWs manage multiple conditions simultaneously. Second, there is a wide implementation gap, i.e. despite promising results from trials, many national CHW programs across the world are yet to have formal roles in NCD management. For example, in Nepal, official roles of FCHVs are still limited to maternal and child health services as per the country’s policy. Therefore, there is a limited understanding of how task-sharing with CHWs can be translated from research to practice, implemented in the real world and sustained over time. Third, there is no study testing the effectiveness of SMS messages for NCDs in community settings in Nepal. Previous studies were done in the outpatient hospital settings only, who may have greater range of comorbidities compared to community population.

### Objectives {7}

The primary objective of this trial is to determine the effectiveness of a packaged intervention containing task-sharing with FCHVs and SMS messaging for management of hypertension, type 2 diabetes, and tobacco smoking on change in systolic blood pressure, fasting blood glucose and smoking cessation, respectively. The secondary objective is to identify demographic and socio-economic factors associated with greater change in systolic blood pressure, fasting blood glucose, and/or smoking cessation.

### Trial design {8}

This is an investigator-initiated, pragmatic, superiority, parallel group (1:1), open-label cluster randomized controlled (c-RCT) trial embedded in a type 2 hybrid effectiveness-implementation research study. While the project contains a qualitative study and an implementation study before and after the trial respectively, this paper describes the protocol of the trial only.

## Methods: Participants, interventions and outcomes

### Study setting {9}

**Figure 1:**
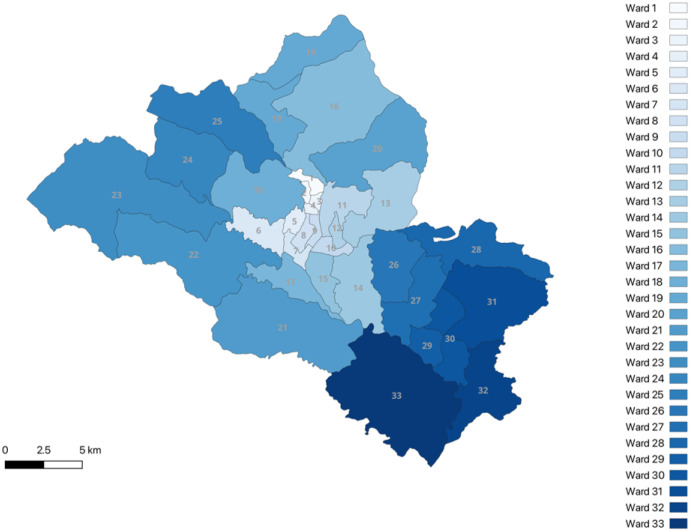
Map of the Pokhara Metropolitan City area

The study will be conducted in Pokhara Metropolitan City, the second most populous city in Nepal after Kathmandu. According to the Census 2021, there are a total of 513,504 residents, of whom 51.8% are female; 19.2% are aged 50 years or older; 88.7% are literate; and 25% of adults work in agriculture.^31^ Pokhara Metropolitan City is divided into 33 administrative units known as “wards” (Figure 1), which will be treated as clusters in this trial. Health services are provided through the following major three systems: the governmental health system, private-owned health system, and NGO or charity-based health services. Within the governmental health system, there are four major tiers: (1) FCHVs provide essential health services through door-to-door home visits. As per the national policy, FCHVs have official roles in maternal and child health services, with no formal responsibilities for NCD management; (2) At health posts (or urban health centres), health assistants or auxiliary health workers can measure BP using digital BP monitors and blood glucose using capillary blood glucose monitors. They can also refill certain oral antihypertensive and hypoglycaemic medicines prescribed by doctors; (3) At least one doctor and a few nurses are available in primary health centres, which usually manage ambulatory cases only. Here, patients can be diagnosed by doctors and initiate or titrate their pharmacological treatments, including getting insulin and other injectable medicines; (4) At the hospitals, doctors with subspecialities are available, where patients can be referred for uncontrolled cases or hospitalized due to severe complications. They can also start pharmacological treatment for smoking cessation. In Pokhara Metropolitan City, there are 656 FCHVs, 26 health posts/urban health centres, two primary health centres, and one district hospital.

### Eligibility criteria {10}

An individual will be included if 1) they are between 40 and 75 years of age, 2) an individual him/herself or their family member own a mobile phone, and 3) have at least one of the following three conditions: hypertension, diabetes, or current tobacco smoking. Hypertension will be defined as the mean of the second and third blood pressure readings ≥ 140/90 mm Hg at both of the two separate occasions, which occurs within a week, regardless of medication status. Diabetes will be defined as fasting finger-prick glucose ≥ 100mg/dL, fasting plasma glucose ≥ 126 mg/dL, and HbA1c ≥6.5%, regardless of medication status. Current tobacco smoking will be defined as having smoked more than 100 cigarettes in their lifetime and currently smoking daily. An individual also needs to be a registered voter in Pokhara Metropolitan City.

**Figure 2:**
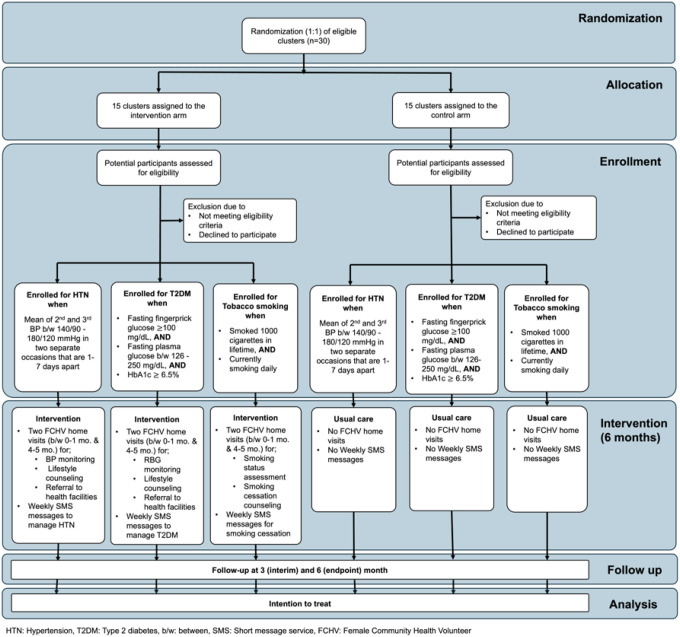
CONSORT flow chart

An individual will be excluded if meeting any of the following conditions: 1) hypertensive urgency or emergency defined as the mean of the second and third blood pressure readings ^3^180/120 mmHg, 2) hyperglycaemia defined as fasting plasma glucose >250 mg/dL, 3) hypoglycaemia defined as fasting plasma glucose <54 mg/dL, 4) diagnosed with secondary hypertension by a healthcare professional, 5) diagnosed with any diabetes other than type 2, 5) terminally ill with a life expectancy of less than six months as determined by a healthcare professional, 6) presenting with acute symptoms (i.e. nausea, vomiting) necessitating immediate medical evaluation, 7) pregnant or planned to be pregnant during the study period, 8) planned to migrate outside of the ward where s/he lives during the study period, 9) using combined estrogen-progestin contraceptives, 10) having a pacemaker or other implanted cardiac device. Interventions are to be delivered by FCHVs who have an unexpired certificate issued by the government and are actively providing services in assigned communities. The CONSORT flow chart is presented below (Figure 2)

### Who will take informed consent? {26a}

Informed consent will be obtained by trained research staff, who are fluent in Nepali (the local language), and have completed an undergraduate program related to health or social sciences. They will participate in a seven-day training programme on ethical practices for human subject research, participant recruitment, screening and consent procedures, and data entry using REDCap. Consent discussions will occur at homes of potential participants, during which trained research staff will present the consent form and obtain signatures if agreed to participate. All participants will receive copies of the signed consent forms. There will be two separate consent procedures in this study. The first is a brief consent for screening, administered to individuals who meet the age criterion of 40–75 years. The second is a full enrollment consent, administered to participants who meet all eligibility criteria, including having at least one of the following conditions: hypertension, diabetes, and/or current tobacco smoking.

### Additional consent provisions for collection and use of participant data and biological specimens {26b}

N/A as there are no ancillary studies planned.

## Interventions

### Explanation for the choice of comparators {6b}

The clusters assigned to the control arm (i.e., comparator) will receive usual care, which reflects standard health services. Under usual care, a participant will not receive home visits by FCHVs to address his or her chronic conditions such as hypertension, diabetes and tobacco smoking. FCHVs may still visit his or her house if there is a periconceptual or pregnant woman, a mother, or a child under five years old in the same household. However, as addressed earlier, their services will be limited to sexual, reproductive, maternal, neonatal, and child health services and will not be extended to chronic conditions such as hypertension, diabetes, and tobacco smoking.

### Intervention description {11a}

Clusters will be randomly assigned to either the intervention arm or the usual care arm for a duration of six months. Participants in the intervention arm will receive a package of intervention named “SCALE-NCD,” which consists of two key components.

The first component is regular FCHV home visits to address hypertension, diabetes, and/or tobacco smoking. Trained FCHVs will visit homes of participants in the intervention arm twice during the trial. Within a month from randomization, participants in the intervention arm will be exposed to the first FCHV visit, during which FCHVs will measure blood pressure, blood glucose, and assess smoking status of participants. FCHVs will also provide motivational-interview-based lifestyle counselling focusing on healthy diet, limiting salt intake, promoting weight loss and physical activities, avoiding harmful alcohol consumption, smoking cessation, and regular blood pressure and/or blood glucose monitoring. The health counselling sessions will not only focus on individual-level risk factors but will also explore structured barriers that may hinder optimal management. For instance, if a participant is unaware of the availability of free antihypertensive medications at primary health centres, FCHVs will provide that information and guide them to access these services. Similarly, if a participant faces challenges in finding a suitable space for physical exercise, FCHVs will offer advice on publicly available recreational centres to support their needs. They will also evaluate medication adherence and refer high-risk individuals to doctors’ office for further assessment. Participants will be exposed to the second FCHV home visit between 4- and 5-months post-randomization, where the contents will be the same as for the first visit.

FCHVs working in intervention clusters will be invited to a 5-day training as these tasks are beyond their routine practices. Trainings will include didactic lectures on risk factors and complications related to hypertension, diabetes, and tobacco smoking, as well as practical sessions focusing on motivational interviews to facilitate behavioural changes (including smoking cessation), blood pressure measurements using digital BP monitors, and blood glucose measurements using glucometers. Only those who successfully pass the final assessment and provided consent will deliver these services in their assigned clusters.

Another component is mobile phone messages. Participants in the intervention arm will receive messages through short message service (SMS) to their mobile phones on a regular basis. The messages will include general health education content, such as advice on maintaining a healthy diet, engaging in physical activity, quitting smoking, and avoiding the harmful use of alcohol, maintaining a healthy weight, and adhering to prescribed treatment. Message contents will be tailored according to each participant’s risk profile. Messages will be sent once a week between 9–10 am, in a 160-character format, based on findings from the qualitative study and co-designing workshop conducted during the first phase of the project and based on our previous pilot study^32^. For participants who are unable to read or do not have a mobile phone, messages will be sent to a participant-nominated family member’s phone. This intervention is designed to maintain healthy behaviours that participants are able to achieve through home counselling by FCHVs. Table 1 below details the components of the intervention.

### Criteria for discontinuing or modifying allocated interventions {11b}

Criteria for modifying allocated interventions will be overseen by a study physician based in Pokhara Metropolitan City, who will be hired specifically for this study. The physician will be accessible 24/7 via a direct phone line throughout the study period to address any concerns that arise from research staff or FCHVs. Additionally, the physician will conduct meetings with FCHVs in the intervention clusters every four months to review any issues identified during home visits. Should the study physician raise any concerns regarding the screening or counselling provided by the FCHVs, the FCHVs will contact the relevant participants to modify their management plan in accordance with the physician’s recommendations.

### Strategies to improve adherence to interventions {11c}

To monitor and improve adherence to the intervention, multiple strategies will be implemented. The importance of fidelity will be explained to FCHVs during the training sessions. FCHVs will carry and fill out a paper-based register documenting each home visit. Field supervisors will review these registers regularly for completeness and provide feedback as needed. The data from the registers will be entered into an electronic database developed in REDCap to enable monitoring. In addition, during the midline and endline evaluation at 3 and 6 months, respectively, participants in the intervention arm will be asked the number of FCHV visits they received. These self-reports will be triangulated with FCHV records to assess fidelity and adherence to scheduled visits. These combined strategies will help ensure that the intervention is delivered as intended and allow timely identification of implementation gaps.

### Relevant concomitant care permitted or prohibited during the trial {11d}

Concomitant care and other interventions will be permitted during the trial. Participants in both the intervention and control arms will be free to seek any care from any health facilities as needed. This approach reflects real-world conditions and supports the external validity of the trial.

### Provisions for post-trial care {30}

To support continuity of care beyond the trial period, the study team will coordinate with Pokhara Metropolitan City to develop a phase-in plan for integrating the SCALE-NCD intervention into the existing health system. All medical devices provided to FCHVs, including digital blood pressure monitors and glucometers, will not be withdrawn at the end of the study. FCHVs will be encouraged to continue providing services for those with hypertension, diabetes, or tobacco smoking after the trial concludes.

### Outcomes {12}

Primary outcomes are mean change SBP (mmHg), fasting plasma glucose (mg/dL), and 30 days tobacco abstinence (yes/no). Secondary outcomes will include change in mean diastolic blood pressure (mmHg), blood pressure control (<140/90 mmHg) (yes/no), HbA1c (%), weight (kg), total cholesterol (mg/dL), high-density lipoprotein (HDL) cholesterol (mg/dL), low-density lipoprotein (LDL) cholesterol (mg/dL), total glycerides (mg/dL), adherence to antihypertensive medicines (yes/no), adherence to hypoglycaemic medicines (yes/no), Fagerstrom Test for Nicotine Dependence (FTND) score, 7-days tobacco abstinence (yes/no), harmful alcohol consumption (yes/no), low salt intake (yes/no), high physical activity (yes/no), high fruits and vegetable intake (yes/no).

### Participant timeline {13}

**Figure 3:**
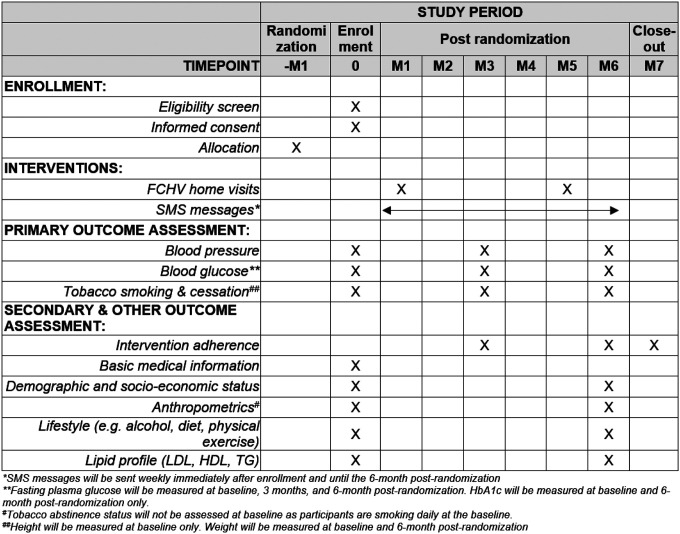
Study timeline

The intervention will consist of six months of either the SCALE-NCD intervention or usual care. In the intervention arm, participants will receive FCHVs home visits twice, while receiving SMS messages weekly. Study team visits for follow-up data collection will occur at 3 months and 6 months. A schematic diagram is presented in Figure 3.

### Sample size {14}

We calculated the required sample size to achieve 90% power for each of the three hypotheses using PASS 2022 software (v22.0.4), with formulations derived from Campbell and Walters (2014)^33^ and Ahn, Heo, and Zhang (2015)^34^. Sample size was calculated for each outcome using a Bonferroni-adjusted method, and the largest required sample size was selected to ensure adequate power for all outcomes. Given the multiple hypotheses corresponding to three different outcomes, we adjusted for multiple comparisons using a Bonferroni correction and set the significance level at 0.05/3 = 0.017 for each test.

For the fasting blood glucose sample size calculation, we assumed a coefficient of variation (COV) of the cluster size of 0.65, a standard deviation of 45.5^27^ and an intra-class correlation coefficient (ICC) of 0.01.^27^ We determined that sample sizes of 90 in the intervention group and 90 in the control group, obtained by sampling 15 clusters with an average of 6 subjects each, were required to detect a difference between the group means of at least −26 mg/dl. After adjusting for a 15% attrition rate, the participant enrollment from each cluster is 9/0.85 = 7, resulting in a sample size of 105 per arm.

For SBP, we assumed the same COV and ICC as 0.01, with a standard deviation of 15.46.^26^ An average of 23 subjects per cluster per arm would achieve 90% power to detect a difference between the group means of at least −5 mmHg. After adjusting for a 15% attrition rate, the participant enrollment from each cluster is 23/0.85 = 27, resulting in a sample size of 405 per arm.

For smoking cessation, we used the test for two proportions in c-RCTs, with formulations from Donner and Klar (2000). We assumed an ICC of 0.01 and a proportion of smoking quitters in the control group of 0.019.^28^ Sample sizes of 450 per arm, obtained by sampling 15 clusters with 30 subjects each, achieve 80% power to detect a difference between the group proportions of 0.05. After accounting for a 15% dropout rate, we require 35 participants in each group, resulting in a total sample size of 525 per group.

Using Bonferroni-adjustment for each outcome and taking the largest sample size is conservative and may overestimate requirements. Despite the outcomes being correlated, this univariate approach ensures adequate power and simplifies interpretation without assuming a multivariate distribution.

### Recruitment {15}

Recruitment will be conducted by trained research staff across 30 clusters in Pokhara Metropolitan City. A sampling framework of all individuals between age 40 and 75 years old in Pokhara Metropolitan city will be identified from the publicly available voter list of the Nepal Election Commission (2025). Then they will be stratified by voting centre (total voting centre = 98), sex (male or female), and four age categories (40–48, 49–57, 58–66, 67–75), totalling 784 (=98*2*4) strata. Individuals will be randomly selected within each stratum using computer generated random numbers. Research staff will visit the homes of selected individuals to provide information about the study. If unavailable, staff will attempt to revisit over the subsequent two days. If an individual remains unavailable after three consecutive visits, research staff will skip that household and proceed to the next.

## Assignment of interventions: allocation

### Sequence generation {16a}

Randomization will be conducted at the cluster level, treating wards in Pokhara Metropolitan city as a unit of analysis. Since there are odd number of wards in Pokhara Metropolitan City, three small wards (wards 1, 2, and 3) and two small wards (wards 4 and 5) will be combined respectively, forming a total of 30 clusters (independent 28 wards and 2 combined wards). These total of 30 clusters will be randomly assigned to either the intervention or the control arm in a 1:1 ratio. Randomization of clusters will be performed using stratified permuted block randomization, stratified by population size of wards. Wards with less than 10,000 people, between 10,000 and 30,000 people, and greater than 30,000 people are categorized to small, medium, and large, respectively. Three sequences were created by choosing blocks of size 2 (AB) and 4 (AABB) with equal probability. The resulting blocks were permuted at random and then concatenated.

### Concealment mechanism {16b}

Concealment will be performed by having an independent biostatistician from Johns Hopkins Biostatistics Centre execute randomization, who is not involved in this project.

### Implementation {16c}

Using the allocation sequence developed by an independent biostatistician, 30 clusters will be assigned to either the intervention or the control arm. When research staff recruit a participant from an intervention cluster, the coordinating centre in Nepal will notify a trained FCHV working in the respective cluster, who will visit the home of the participant within a month from the enrollment. The coordinating centre will also subsequently notify a SMS message company in Nepal, so that text messages will be scheduled and sent to the participant between 9–10 am on weekly basis. Research staff will recruit a participant in control clusters with the same approach, where the coordinating centre will notify neither FCHVs nor the SMS company.

## Assignment of interventions: Blinding

### Who will be blinded {17a}

The senior statistician overseeing the project will be blinded. Trial participants and care providers (i.e. FCHVs) will not be blinded due to the nature of the intervention. Investigators and other staffs who support the coordination centres in Nepal on the daily basis, including the PI, will not be blinded.

### Procedure for unblinding if needed {17b}

N/A as the senior statistician is the only one blinded.

## Data collection and management

### Plans for assessment and collection of outcomes {18a}

In the trial, data will be collected at three time points: baseline (0 months), interim (3 months), and endline (6 months). The types of data collected and its specific timeline has been presented earlier. In addition to data for primary and secondary outcomes, we will collect data for intervention adherence, which will be used for sensitivity analysis. To maintain data quality, several procedures will be undertaken. First, data collection will be entirely digitalized to minimize data entry errors. Trained research staff will enter data directly into the REDCap application on tablets provided by the coordinating center. Each morning, the tablets will be distributed to the research staff, and all devices will be collected back at the end of the day, when staff in coordinating centers will check the condition of tablets and update the operating systems if needed. Data entered by research staff will be monitored daily by the data management team, which is comprised of data managers in the coordinating center and one data manager in Johns Hopkins Bloomberg School of Public Health. Any duplications, missing values, or other data entry errors will be reported to a senior staff member at the coordinating center, who will address the issues by communicating with the field research staff and, when necessary, the investigators. Second, research staff will be trained for data collection techniques through 7-day training prior to the field placement, during which they will be trained on the use of REDCap, verbatim questionnaire administrations, and clinical assessment techniques (e.g. BP measurements, finger prick glucose measurement, height, weight).

Study instruments will be pre-tested with population living outside of the study site to ensure clarity, user-friendliness, and consistency in interpretation among participants.

### Plans to promote participant retention and complete follow-up {18b}

To promote participant retention, research staff will make a phone call to participants the day before each follow-up visit to remind them of the scheduled visit. If there is a participant drop out, we will identify the reason why (i.e. deaths, participant withdrew informed consent, migration) and compare the baseline characteristics of those who dropped out vs retained in the study to explore the extent of the selection bias. For those remained in the study but deviated from the intervention protocol, all outcome data will be collected and used for sensitivity analysis (i.e. exploratory per-protocol analysis)

### Data management {19}

Data will be stored in Johns Hopkins University (JHU) REDCap, which is HIPPA compliant and approved by the Johns Hopkins Bloomberg School of public Health (JHSPH) Institutional Review Boards (IRB). Only authorized personnel with username and password can access the JHU REDCap database, and each of their access will be logged and recorded to maintain accountability. All personally identifiable data will be deleted before data transfer, and the original dataset (i.e., containing personally identifiable data) will be erased five years after the study’s completion. Tablets used for data collection will be encrypted while the data is on storage devices. These devices will be routinely updated with security patches. Measures to maintain data quality is described earlier.

### Confidentiality {27}

As addressed earlier, personally identifiable information of enrolled participants will be stored on encrypted devices. Only authorized personnel will have access to the JHU REDCap database, whose access will be recorded. All identifiable information will be de-identified before sharing or publication.

### Plans for collection, laboratory evaluation and storage of biological specimens for genetic or molecular analysis in this trial/future use {33}

Laboratory technicians certified in Nepal will collect 5 mL of venous blood sample at the baseline and endline from all participants. HbA1c and lipid panels (total cholesterol, LDL cholesterol, HDL cholesterol, and triglyceride) will be assessed for all participants while fasting plasma glucose will be additionally measured for those who had fasting fingerprick glucose ≥100 mg/dL. All samples will be analyzed following the reference method approved by the International Federation of Clinical Chemistry and Laboratory Medicine (IFCC). All specimens will be properly disposed of after completion of tests. No genetic or molecular analyses are planned for this trial or any ancillary studies.

## Statistical methods

### Statistical methods for primary and secondary outcomes {20a}

The primary analysis will follow an intention-to-treat approach, comparing the mean differences in changes in systolic blood pressure, fasting plasma glucose levels, and changes in smoking status between individuals assigned to the intervention arm vs control arms. Primary outcomes will be measured at baseline and six months, requiring models that account for repeated measures and the hierarchical structure of participants nested within sites. To compare changes between groups, we will use mixed-effects models for each outcome: linear mixed-effects models for continuous outcomes (SBP, fasting blood glucose) and a mixed-effects logistic regression for the binary outcome (smoking status), each including a random intercept for participants. Fixed effects will include intervention, time (baseline and 6 months), their interaction, and potential confounders. For continuous outcomes, the interaction term represents the difference in mean change between groups. For the binary outcome, it represents the difference in change in log-odds of smoking cessation, with the odds ratio indicating how much greater (or smaller) the change in odds of cessation is for the intervention group relative to control.

Although outcomes may be correlated, the univariate approach is conservative and simplifies interpretation without assuming a multivariate distribution. Sensitivity analyses will use multivariate models accounting for correlations between outcomes. Continuous outcomes will be analyzed jointly using repeated-measures MANOVA or a multivariate linear mixed model with an unstructured covariance matrix. For mixed outcome types, a multivariate logistic mixed model or joint modeling approach will be applied. These models account for both outcome correlations and data hierarchy, allowing estimation of overall intervention effects and assessment of robustness relative to univariate analyses.

### Interim analyses {21b}

Interim analysis for efficacy will be done at 3 months. Given the low-risk nature of this study, there will be no stopping guidelines established.

### Methods for additional analyses (e.g. subgroup analyses) {20b}

Exploratory subgroup analyses will be conducted by sex, age categories, socioeconomic status (including education level), hypertension stage, HbA1c stage, and smoking level. Analyses will be stratified if effect modification is observed and adjusted for potential confounders. Additional exploratory analyses may be conducted as appropriate based on emerging findings.

### Methods in analysis to handle protocol non-adherence and any statistical methods to handle missing data {20c}

Exploratory sensitivity analysis will be performed by comparing analyses conducted on intention-to-treat population vs per-protocol (i.e. adherent) population. For any variable in the final dataset that has more than 5% missing values, a series of sensitivity analyses will be performed, such as comparing analyses conducted on complete cases vs those conducted using an appropriate imputation framework.

### Plans to give access to the full protocol, participant level-data and statistical code {31c}

The full study protocol, de-identified participant-level dataset, and statistical code will be made available by the corresponding author upon reasonable request and subject to approval by the study team and relevant committees.

## Oversight and monitoring

### Composition of the coordinating center and trial steering committee {5d}

The coordinating center will be established in Pokhara Metropolitan City, Nepal, at the Nepal Development Society (NeDS) Pokhara office. It will be responsible for training research staff and FCHVs, supporting data management and responding to any safety concerns (e.g., adverse events) arising from study sites. The coordinating center will be served by full-time NeDS officers with bachelor’s or higher degrees in health science related fields. The data management team will be jointly comprised by a few data managers in the coordinating center as well as a data manager in JHSPH. Endpoint adjudication will not be performed. An independent Technical Advisory Board (TAB) will also be established, comprising of a diabetologist, cardiologist, public health researcher, health policy expert, and community health experts, which will provide overall supervision of the trial, ensuring the trial is conducted to high scientific, ethical, and regulatory standards. The TAB will meet once before the enrolment and twice during the trial. Day-to-day trial operations will be managed through regular meetings between the coordinating centre and JHSPH, held at least weekly and more frequently as needed.

### Composition of the data monitoring committee, its role and reporting structure {21a}

An independent Data Safety Monitoring Board (DSMB) will be established, comprising of an epidemiologist, biostatistician, cardiologist, and public health experts. The DSMB will be responsible for monitoring participant safety and the integrity of trial data, with a focus on detecting harm or benefit early. The board will be given an unblinded and deidentified dataset upon request to the PI and will receive all reporting on adverse events and serious adverse events, using a reporting template approved by the DSMB themselves.

The DSMB will convene a total of three times; before the enrolment, half-way through the trial, and at the endline of the trial.

### Adverse event reporting and harms {22}

Any unanticipated adverse or serious adverse events to participants will be reported to the JHSPH IRB, Nepal Health Research Council (NHRC) IRB, and the DSMB.

### Frequency and plans for auditing trial conduct {23}

Formal auditing procedures will not be conducted for this trial.

### Plans for communicating important protocol amendments to relevant parties (e.g. trial participants, ethical committees) {25}

Any major protocol amendments will be reported to clinicaltrial.gov registration and submitted for approval to both the JHSPH IRB and the NHRC IRB prior to implementation. Once approved, such changes will be communicated with trial participants by research staff in Nepal.

### Dissemination plans {31a}

The trial findings will be disseminated to scientific communities via peer-reviewed journal publications and conference presentations. They will also be disseminated to community members (including participants), health care professionals, and policymakers in Nepal via dissemination meetings and other platforms such as press conference and media outreach in Nepal.

## Discussion

To the best of our knowledge, this is the first trial to evaluate the effectiveness of task-sharing with CHWs in managing multiple cardiovascular disease (CVD) risk factors concurrently—hypertension, diabetes, and tobacco use—at scale in an urban LMIC setting. While CHWs have played a pivotal role in rural primary health care systems, their potential to deliver integrated NCD services in urban settings remains largely underexplored.

This trial offers important new insights by shifting the geographic and contextual focus of CHW-led interventions to urban areas. Most prior task-sharing studies involving CHWs have been conducted in rural LMIC settings, where they often served as the sole point of access to essential health services. However, urban populations present distinct challenges and opportunities—such as competing providers from non-governmental healthcare systems, high population density, and a wider range of health risks, including greater exposure to ultra-processed food and sedentary lifestyles. These conditions introduce greater variability in both individual-level health needs and health system-level coordination, which must be accounted for when designing and evaluating public health interventions. By embedding this trial in Pokhara Metropolitan City, a rapidly urbanizing metropolitan city in Nepal, this study will help assess whether task-sharing with CHWs remains effective in more complex, densely populated health system contexts.

This study also advances the implementation science field methodologically. Unlike earlier trials conducted by our team in peri-urban Nepal, which demonstrated the effectiveness of sharing tasks with CHWs in managing hypertension, diabetes, and smoking cessation separately, this trial adopts a more realistic, integrated approach. CHWs will screen, counsel, and refer high risk individuals with any of these three conditions during home visits. This mirrors how CHWs are increasingly expected to work in real-world settings—efficiently delivering care for multiple chronic conditions during routine visits, in addition to tasks related to other conditions (e.g. maternal and child health) and administrative duties. Thus, this study is designed as an effectiveness trial rather than an efficacy trial, with implementation occurring across a large city and under routine program conditions. As such, it will allow for evaluation of outcomes in a less controlled environment, offering more generalizable evidence. This transition from tightly monitored settings to broader program conditions is essential for understanding how task-sharing with CHWs can function when scaled through routine government platforms.

A further strength of this trial is its integration within a type 2 hybrid effectiveness-implementation research study design. Most CHW trials to date have focused solely on assessing clinical outcomes without systematically evaluating how interventions were delivered, how acceptable and appropriate interventions were, and how sustainable interventions could be. Our study is designed to simultaneously examine both effectiveness and implementation outcomes—including acceptability, appropriateness, reach, adoption, fidelity, implementation, sustainability, and cost—using the Consolidated Framework for Implementation Research (CFIR) and its Outcomes Addendum framework. These implementation outcomes are critical for informing the scalability of integrated NCD care models through CHWs in Nepal and similar LMIC settings. It will allow us to estimate population-level impact by assessing reach and effectiveness in the realistic settings and also help identify contextual factors that influence implementation success, which is particularly important for interventions operating across diverse urban neighborhoods. This study will provide policymakers with actionable data on the value and scalability of integrated CHW-led models for urban NCD control in Nepal and similar settings. In addition, this trial incorporates a digital health intervention, including weekly text messages tailored to participants’ risk profiles. These mHealth strategies aim to reinforce CHW-delivered counseling and improve adherence to lifestyle changes or medication. The use of mobile phones as an adjunct to task-sharing could offer a scalable, low-cost tool to support behavior change in LMICs, particularly in urban populations with growing mobile penetration. This design acknowledges the realities of resource-limited health systems, where physician shortages and resource constraints necessitate innovative models for essential health care delivery.

Finally, beyond clinical effectiveness, this trial will generate important operational insights. These include strategies for recruiting and retaining CHWs in urban areas, training them to manage multiple NCDs simultaneously, and sustaining motivation through supervision, incentives, and integration within the health system. It will also shed light on challenges such as navigating private sector dynamics, engaging hard-to-reach populations, maintaining data quality across a larger geographic area, and leveraging digital tools for behavior change communication. The SCALE-NCD study will not only generate robust evidence on effectiveness and implementation outcomes but also guide future adaptations and policy-level decisions for community-based NCD programs.

## Figures and Tables

**Table 1:** Key components of SCALE NCD interventions and the FCHV training

Key components of SCALE-NCD intervention	
**FCHV home visit** First visit between 0–1 month. Second visit between 4–5 months For 30 mins/visit	**Screening and measurements** Resting BP (digital BP devices) Fasting blood glucose (finger prick) Smoking status assessment
	**Brief motivational interview-based health counseling** Healthy diet Salt intake reduction (<5g/day) o Higher vegetable and fruits intake Weight loss (BMI within 18.5–24.9 kg/m2) Physical exercises (mid-high intensity >150 min/week) Avoidance of harmful alcohol intake (<1 standard drink/day) Smoking cessation Identification and approach to social determinants
	**Medication adherence** Current adherence status
	**Referral of high-risk individuals** BP ≥140/90 mmHg Finger prick blood glucose ≥126 mg/dL
**Mobile phone messages** Once/wk. 10 am	**Individually tailored health education** Advice on healthy diet, exercise, smoking cessation, harmful use of alcohol

## Data Availability

The final trial dataset will be made available as appropriate from the corresponding author on reasonable request.

## Abbreviations

BP: Blood Pressure
CVD: Cardiovascular Disease
c-RCT: Cluster Randomized Controlled Trial
COV: Coefficient of Variation
CHW: Community Health Worker
CFIR: Consolidated Framework for Implementation Research
DSMB: Data and Safety Monitoring Board
FCHV: Female Community Health Volunteer
HIPAA: Health Insurance Portability and Accountability Act
HbA1c: Hemoglobin A1c
HDL: High-density Lipoprotein
IRB: Institutional Review Board
ICC: Intra-class Correlation Coefficient
JHSPH: Johns Hopkins Bloomberg School of Public Health
LMIC: Low- and Middle-income Country
LDL: Low-density Lipoprotein
NeDS: Nepal Development Society
NHRC: Nepal Health Research Council
NCD: Non-communicable Disease
NGO: Non-governmental Organization
REDCap: Research Electronic Data Capture
SMS: Short Message Service
SBP: Systolic Blood Pressure
TAB: Technical Advisory Board
WHO: World Health Organization
